# Supplementary material for: Journal policies and editors’ opinions on peer review
Source: eLife. 2020 Nov 19;9:e62529. doi: 10.7554/eLife.62529 (PMC7717900; doi:10.7554/eLife.62529)
Supplement: Supplementary file 2. [file elife-62529-supp2.docx]

# SUPPLEMENTARY FILE 2

Categories used to define disciplines in InCites.

Codebook reliability testing.
