# Supplementary material for: Journal policies and editors’ opinions on peer review
Source: eLife. 2020 Nov 19;9:e62529. doi: 10.7554/eLife.62529 (PMC7717900; doi:10.7554/eLife.62529)
Supplement: Supplementary file 3. [file elife-62529-supp3.docx]

**SUPPLEMENTARY FILE 3**

**Survey Questions**

A1. Could you please indicate your role at [insert journal name]?

- Editor-in-chief (lead editor)
- Deputy editor
- Section editor
- Associate editor
- Managing editor
- Other (please specify)

A2. Does your journal routinely screen manuscripts for plagiarism?

- Always
- Never
- Only if suspicion has been raised
- At editor's discretion
- I don't know
- Other (please specify)

A3. Does your journal ever outsource peer review to a commercial third party?

- Yes
- No
- At editor's discretion
- I don't know
- Other (please specify)

A4. Does your journal offer or use any results-blind peer review pathways? (Select all that apply)

- No
- Protocol review prior to data collection (e.g. 'registered reports')
- Initial blinding of peer reviewers to the results of manuscripts of completed studies (e.g. ‘results-free review’)
- I don't know
- Other (please specify)

A5. Is it policy or routine practice at your journal to allow authors to recommend for or against specific reviewers?

- Yes - Recommend for only
- Yes - Recommend against only
- Yes - Both recommend for and against
- No
- I don't know
- Other (please specify)

A6. With regards to blinding, during peer review do:

|  | A. Authors | B. Peer reviewers | C. Handling editors |
| --- | --- | --- | --- |
| 1. Authors know the identities of | [Not applicable] | - Yes - No - N/A - At reviewer discretion | - Yes - No - N/A - At editor discretion |
| 2. Individual peer reviewers know the identities | - Yes - No - N/A - At author discretion | - Yes - No - N/A - At reviewer discretion | - Yes - No - N/A - At editor discretion |
| 3. Handling editors know the identities of | - Yes - No - N/A - At author discretion | - Yes - No - N/A - At reviewer discretion | [Not applicable] |

A7. Is it policy or routine practice at your journal to allow, or encourage, direct interaction/dialogue between any of the following parties during peer review? (Select all that apply)

- Authors and peer reviewers
- Fellow peer reviewers
- Peer reviewers and the handling editor
- None of the above
- I don't know
- Other (please specify)

A8. When would an editor at your journal be permitted to edit a reviewer's report?

|  | Never acceptable to edit the report | Acceptable to edit the report without reviewer's permission | Acceptable to edit the report, but only with reviewer's permission |
| --- | --- | --- | --- |
| 1. When a reviewer identifies themselves in a blinded peer review framework | ⭘ | ⭘ | ⭘ |
| 2. When the reviewer has used inappropriate or offensive language | ⭘ | ⭘ | ⭘ |
| 3. When the reviewer has made an inappropriate reference to an author's gender, nationality, institution, age etc | ⭘ | ⭘ | ⭘ |
| 4. When there are spelling and/or grammatical errors | ⭘ | ⭘ | ⭘ |
| 5. When the review has English language problems | ⭘ | ⭘ | ⭘ |
| 6. When the reviewer has left in their comments to the editor | ⭘ | ⭘ | ⭘ |
| 7. When the editor disagrees with the reviewer's recommendation | ⭘ | ⭘ | ⭘ |
| 8. Other (please specify): | ⭘ | ⭘ | ⭘ |

A9. What processes would editors at your journal follow if a reviewer's report needed to be edited? (For example, contact the reviewer to discuss the issue, obtain permission to edit, make and show the changes, un-invite the reviewer if permission cannot be obtained etc.)

________________________________________________________________

________________________________________________________________

________________________________________________________________

A10. Does your journal have an official policy on editing peer review reports?

- Yes
- No
- Unsure

A11. Does your journal make any of the following documents available to the readership alongside the published article? (Select all that apply)

- Unsigned peer reviewer reports
- Signed peer review reports
- Author responses
- Editorial decision letters
- None
- I don't know
- Other (please specify)

A12. Upon completion of peer review, what information (if any) is provided to peer reviewers? (Select all that apply)

|  | 1. All peer reviewer reports are | 2. The editorial decision letter is |
| --- | --- | --- |
| Emailed directly to reviewers | □ | □ |
| Available to reviewers for viewing in the submission platform | □ | □ |
| Not shared | □ | □ |
| I don't know | □ | □ |
| Other (please specify) | □ | □ |

A13. What is the journal’s current policy on the availability of research data, materials and code following publication? (Select all that apply)

|  | 1. Research data | 2. Research materials | 3. Research code |
| --- | --- | --- | --- |
| Journal encourages sharing by researchers, but it is not required | □ | □ | □ |
| Journal requires authors to make available post-publication if requested | □ | □ | □ |
| Journal requires an in-text statement to be made as to whether it will be available | □ | □ | □ |
| Journal requires data to be posted to a trusted repository following article publication | □ | □ | □ |
| No policy on availability | □ | □ | □ |
| Not applicable | □ | □ | □ |
| I don't know | □ | □ | □ |
| Other (please specify) | □ | □ | □ |

A14. If you have any other comments you would like to make about the peer review process at the journal you are an editor for, please enter them below

________________________________________________________________

________________________________________________________________

________________________________________________________________

B1. Before we begin, would you please select what discipline most closely represents your journal's field of inquiry?

- Ecology and Evolutionary Biology
- Psychology
- Medicine
- Economics
- Physics/Mathematics/Chemistry

B2. What are your thoughts on co-reviewing? (Invited peer reviewers co-writing reviews with graduate students, junior researchers etc.)

________________________________________________________________

________________________________________________________________

________________________________________________________________

B3. During peer review do you think it is ever appropriate for reviewers to recommend citations of their own work? If so, when? If not, why not?

________________________________________________________________

________________________________________________________________

________________________________________________________________

B4. As an editor would you support, and mediate, a request from a peer reviewer to see a manuscript's raw data if they felt they needed to as part of their review process?

________________________________________________________________

________________________________________________________________

________________________________________________________________

B5. In your view, how often should an editor publish their own original articles in a journal they edit?

________________________________________________________________

________________________________________________________________

________________________________________________________________

B6. What are your thoughts on replication studies? (Studies that follow the methods of another study as closely as possible.)

_______________________________________________________________

________________________________________________________________

________________________________________________________________

B7. What, if anything, would you change about how your journal conducts peer review? (Please include what the current process is that you would change.)

________________________________________________________________

________________________________________________________________

________________________________________________________________
