# Supplementary material for: Journal policies and editors’ opinions on peer review
Source: eLife. 2020 Nov 19;9:e62529. doi: 10.7554/eLife.62529 (PMC7717900; doi:10.7554/eLife.62529)
Supplement: Supplementary file 4. [file elife-62529-supp4.docx]

# SUPPLEMENTARY FILE 4.

# Survey A Responses.

Note: Some proportions will be slightly lower than those presented in the manuscript due to the inclusion of missing response data.

## Question A1 - Could you please indicate your role at the journal?

|  | All | | Ecology | | Economics | | Medicine | | Physics | | Psychology | |
| --- | --- | --- | --- | --- | --- | --- | --- | --- | --- | --- | --- | --- |
|  | N | % | N | % | N | % | N | % | N | % | N | % |
| -99 No response | 3 | 1 | 2 | 2 | 1 | 1 | 0 | 0 | 0 | 0 | 0 | 0 |
| 1 Editor-in-chief (lead editor) | 293 | 91 | 83 | 92 | 68 | 85 | 37 | 92 | 25 | 89 | 80 | 95 |
| 2 Deputy editor | 3 | 1 | 1 | 1 | 1 | 1 | 0 | 0 | 1 | 4 | 0 | 0 |
| 3 Section editor | 2 | 1 | 0 | 0 | 1 | 1 | 1 | 2 | 0 | 0 | 0 | 0 |
| 4 Associate editor | 2 | 1 | 1 | 1 | 1 | 1 | 0 | 0 | 0 | 0 | 0 | 0 |
| 5 Managing editor | 18 | 6 | 3 | 3 | 8 | 10 | 2 | 5 | 1 | 4 | 4 | 5 |
| 6 Other | 1 | 0 | 0 | 0 | 0 | 0 | 0 | 0 | 1 | 4 | 0 | 0 |
|  | 322 |  | 90 |  | 80 |  | 40 |  | 28 |  | 84 |  |

## Question A2 - Does your journal routinely screen manuscripts for plagiarism?

|  | All | | Ecology | | Economics | | Medicine | | Physics | | Psychology | |
| --- | --- | --- | --- | --- | --- | --- | --- | --- | --- | --- | --- | --- |
|  | N | % | N | % | N | % | N | % | N | % | N | % |
| -99 No response | 5 | 2 | 0 | 0 | 2 | 2 | 1 | 2 | 2 | 7 | 0 | 0 |
| 0 Never | 7 | 2 | 4 | 4 | 1 | 1 | 0 | 0 | 1 | 4 | 1 | 1 |
| 1 Always | 154 | 48 | 46 | 51 | 24 | 30 | 25 | 62 | 14 | 50 | 45 | 54 |
| 2 Only if suspicion has been raised | 84 | 26 | 24 | 27 | 30 | 38 | 4 | 10 | 7 | 25 | 19 | 23 |
| 3 At editor's discretion | 54 | 17 | 14 | 16 | 18 | 22 | 5 | 12 | 4 | 14 | 13 | 15 |
| 4 I don't know | 5 | 2 | 0 | 0 | 2 | 2 | 0 | 0 | 0 | 0 | 3 | 4 |
| 5 Other | 13 | 4 | 2 | 2 | 3 | 4 | 5 | 12 | 0 | 0 | 3 | 4 |
|  | 322 |  | 90 |  | 80 |  | 40 |  | 28 |  | 84 |  |

## Question A3 - Does your journal ever outsource peer review to a commercial third party?

|  | All | | Ecology | | Economics | | Medicine | | Physics | | Psychology | |
| --- | --- | --- | --- | --- | --- | --- | --- | --- | --- | --- | --- | --- |
|  | N | % | N | % | N | % | N | % | N | % | N | % |
| -99 No response | 4 | 1 | 0 | 0 | 2 | 2 | 0 | 0 | 0 | 0 | 2 | 2 |
| 0 No | 315 | 98 | 90 | 100 | 78 | 98 | 38 | 95 | 27 | 96 | 82 | 98 |
| 1 Yes | 2 | 1 | 0 | 0 | 0 | 0 | 1 | 2 | 1 | 4 | 0 | 0 |
| 2 At editor's discretion | 0 | 0 | 0 | 0 | 0 | 0 | 0 | 0 | 0 | 0 | 0 | 0 |
| 3 I don't know | 0 | 0 | 0 | 0 | 0 | 0 | 0 | 0 | 0 | 0 | 0 | 0 |
| 4 Other | 1 | 0 | 0 | 0 | 0 | 0 | 1 | 2 | 0 | 0 | 0 | 0 |
|  | 322 |  | 90 |  | 80 |  | 40 |  | 28 |  | 84 |  |

## Question A4 - Does your journal offer or use any results-blind peer review pathways?

|  | All | | Ecology | | Economics | | Medicine | | Physics | | Psychology | |
| --- | --- | --- | --- | --- | --- | --- | --- | --- | --- | --- | --- | --- |
|  | N | % | N | % | N | % | N | % | N | % | N | % |
| -99 No response | 12 | - | 5 | - | 4 | - | 1 | - | 1 | - | 1 | - |
| 0 No | 237 | 76 | 75 | 88 | 59 | 78 | 30 | 77 | 19 | 70 | 54 | 65 |
| 1 Registered reports | 24 | 8 | 2 | 2 | 2 | 3 | 4 | 10 | 0 | 0 | 16 | 19 |
| 2 Results-free review | 19 | 6 | 1 | 1 | 8 | 11 | 2 | 5 | 1 | 4 | 7 | 8 |
| 3 I don't know | 18 | 6 | 5 | 6 | 5 | 7 | 1 | 3 | 4 | 15 | 3 | 4 |
| 4 Other | 16 | 5 | 2 | 2 | 3 | 4 | 3 | 8 | 3 | 11 | 5 | 6 |
| Total responses | 310 | - | 85 | - | 76 | - | 39 | - | 27 | - | 83 | - |

*Multiple answers possible - Percentages will not add up to 100%

## Question A5 - Is it policy or routine practice at your journal to allow authors to recommend for or against specific reviewers?

|  | All | | Ecology | | Economics | | Medicine | | Physics | | Psychology | |
| --- | --- | --- | --- | --- | --- | --- | --- | --- | --- | --- | --- | --- |
|  | N | % | N | % | N | % | N | % | N | % | N | % |
| -99 No response | 1 | 0 | 0 | 0 | 1 | 1 | 0 | 0 | 0 | 0 | 0 | 0 |
| 0 No | 73 | 23 | 0 | 0 | 47 | 59 | 4 | 10 | 4 | 14 | 18 | 21 |
| 1 Yes - Recommend for only | 27 | 8 | 11 | 12 | 2 | 2 | 4 | 10 | 4 | 14 | 6 | 7 |
| 2 Yes - Recommend against only | 12 | 4 | 3 | 3 | 3 | 4 | 1 | 2 | 0 | 0 | 5 | 6 |
| 3 Yes - Both recommend for and against | 197 | 61 | 75 | 83 | 23 | 29 | 29 | 72 | 19 | 68 | 51 | 61 |
| 4 I don't know | 0 | 0 | 0 | 0 | 0 | 0 | 0 | 0 | 0 | 0 | 0 | 0 |
| 5 Other | 12 | 4 | 1 | 1 | 4 | 5 | 2 | 5 | 1 | 4 | 4 | 5 |
|  | 322 |  | 90 |  | 80 |  | 40 |  | 28 |  | 84 |  |

## Question A6 - Journal blinding system*

|  | All | | Ecology | | Economics | | Medicine | | Physics | | Psychology | |
| --- | --- | --- | --- | --- | --- | --- | --- | --- | --- | --- | --- | --- |
|  | N | % | N | % | N | % | N | % | N | % | N | % |
| <NA> Participant left survey | 4 | 1 | 1 | 1 | 0 | 0 | 0 | 0 | 1 | 4 | 2 | 2 |
| -99 No response | 11 | 3 | 2 | 2 | 3 | 4 | 2 | 5 | 1 | 4 | 3 | 4 |
| 0 Open identities | 3 | 1 | 0 | 0 | 1 | 1 | 2 | 5 | 0 | 0 | 0 | 0 |
| 1 Single-blind | 176 | 55 | 68 | 76 | 33 | 41 | 33 | 82 | 26 | 93 | 16 | 19 |
| 2 Single-blind (hybrid) | 12 | 4 | 3 | 3 | 1 | 1 | 0 | 0 | 0 | 0 | 8 | 10 |
| 3 Double-blind (hybrid) | 4 | 1 | 1 | 1 | 0 | 0 | 0 | 0 | 0 | 0 | 3 | 4 |
| 4 Double-blind | 109 | 34 | 15 | 17 | 40 | 50 | 3 | 8 | 0 | 0 | 51 | 61 |
| 5 Triple-blind | 3 | 1 | 0 | 0 | 2 | 2 | 0 | 0 | 0 | 0 | 1 | 1 |
|  | 322 |  | 90 |  | 80 |  | 40 |  | 28 |  | 84 |  |

*Refer to “PRS_Results_Code.R” script for details on how this information was generated

## Question A6_1_2 - During peer review do reviewers know the identity of authors?

|  | All | | Ecology | | Economics | | Medicine | | Physics | | Psychology | |
| --- | --- | --- | --- | --- | --- | --- | --- | --- | --- | --- | --- | --- |
|  | N | % | N | % | N | % | N | % | N | % | N | % |
| <NA> Participant left survey | 4 | 1 | 1 | 1 | 0 | 0 | 0 | 0 | 1 | 4 | 2 | 2 |
| -99 No response | 3 | 1 | 0 | 0 | 1 | 1 | 0 | 0 | 1 | 4 | 1 | 1 |
| 0 No | 113 | 35 | 15 | 17 | 42 | 52 | 3 | 8 | 0 | 0 | 53 | 63 |
| 1 Yes | 186 | 58 | 70 | 78 | 36 | 45 | 37 | 92 | 26 | 93 | 17 | 20 |
| 2 Not applicable | 0 | 0 | 0 | 0 | 0 | 0 | 0 | 0 | 0 | 0 | 0 | 0 |
| 3 At their discretion | 16 | 5 | 4 | 4 | 1 | 1 | 0 | 0 | 0 | 0 | 11 | 13 |
|  | 322 |  | 90 |  | 80 |  | 40 |  | 28 |  | 84 |  |

## Question A6_1_3 - During peer review do editors know the identity of authors?

|  | All | | Ecology | | Economics | | Medicine | | Physics | | Psychology | |
| --- | --- | --- | --- | --- | --- | --- | --- | --- | --- | --- | --- | --- |
|  | N | % | N | % | N | % | N | % | N | % | N | % |
| <NA> Participant left survey | 4 | 1 | 1 | 1 | 0 | 0 | 0 | 0 | 1 | 4 | 2 | 2 |
| -99 No response | 5 | 2 | 1 | 1 | 1 | 1 | 1 | 2 | 1 | 4 | 1 | 1 |
| 0 No | 3 | 1 | 0 | 0 | 2 | 2 | 0 | 0 | 0 | 0 | 1 | 1 |
| 1 Yes | 310 | 96 | 88 | 98 | 77 | 96 | 39 | 98 | 26 | 93 | 80 | 95 |
| 2 Not applicable | 0 | 0 | 0 | 0 | 0 | 0 | 0 | 0 | 0 | 0 | 0 | 0 |
| 3 At their discretion | 0 | 0 | 0 | 0 | 0 | 0 | 0 | 0 | 0 | 0 | 0 | 0 |
|  | 322 |  | 90 |  | 80 |  | 40 |  | 28 |  | 84 |  |

## Question A6_2_1 - During peer review do authors know the identity of reviewers?

|  | All | | Ecology | | Economics | | Medicine | | Physics | | Psychology | |
| --- | --- | --- | --- | --- | --- | --- | --- | --- | --- | --- | --- | --- |
|  | N | % | N | % | N | % | N | % | N | % | N | % |
| <NA> Participant left survey | 4 | 1 | 1 | 1 | 0 | 0 | 0 | 0 | 1 | 4 | 2 | 2 |
| -99 No response | 11 | 3 | 2 | 2 | 3 | 4 | 2 | 5 | 1 | 4 | 3 | 4 |
| 0 No | 249 | 77 | 54 | 60 | 76 | 95 | 32 | 80 | 25 | 89 | 62 | 74 |
| 1 Yes | 3 | 1 | 0 | 0 | 1 | 1 | 2 | 5 | 0 | 0 | 0 | 0 |
| 2 Not applicable | 0 | 0 | 0 | 0 | 0 | 0 | 0 | 0 | 0 | 0 | 0 | 0 |
| 3 At their discretion | 55 | 17 | 33 | 37 | 0 | 0 | 4 | 10 | 1 | 4 | 17 | 20 |
|  | 322 |  | 90 |  | 80 |  | 40 |  | 28 |  | 84 |  |

## Question A7 - Is it policy or routine practice at your journal to allow, or encourage, direct interaction/dialogue between any of the following parties during peer review?

|  | All | | Ecology | | Economics | | Medicine | | Physics | | Psychology | |
| --- | --- | --- | --- | --- | --- | --- | --- | --- | --- | --- | --- | --- |
|  | N | % | N | % | N | % | N | % | N | % | N | % |
| <NA> Participant left survey | 4 | - | 1 | - | 0 | - | 0 | - | 1 | - | 2 | - |
| -99 No response | 4 | - | 0 | - | 1 | - | 0 | - | 0 | - | 3 | - |
| 1 Authors and peer reviewers | 20 | 6 | 7 | 8 | 3 | 4 | 3 | 8 | 4 | 15 | 3 | 4 |
| 2 Fellow peer reviewers | 7 | 2 | 2 | 2 | 1 | 1 | 4 | 10 | 0 | 0 | 0 | 0 |
| 3 Peer reviewers and the handling editor | 230 | 73 | 76 | 85 | 49 | 62 | 31 | 78 | 20 | 74 | 54 | 68 |
| 4 None of the above | 75 | 24 | 12 | 13 | 26 | 33 | 8 | 20 | 6 | 22 | 23 | 29 |
| 5 I don't know | 1 | 0 | 0 | 0 | 1 | 1 | 0 | 0 | 0 | 0 | 0 | 0 |
| 6 Other | 12 | 4 | 4 | 4 | 2 | 3 | 1 | 2 | 1 | 4 | 4 | 5 |
| Total responses | 314 | - | 89 | - | 79 | - | 40 | - | 27 | - | 79 | - |

*Multiple answers possible - Percentages will not add up to 100%

## Question A8_1 - When would an editor at your journal be permitted to edit a reviewer's report?

Situation 1: When a reviewer identifies themselves in a blinded peer review framework.

|  | All | | Ecology | | Economics | | Medicine | | Physics | | Psychology | |
| --- | --- | --- | --- | --- | --- | --- | --- | --- | --- | --- | --- | --- |
|  | N | % | N | % | N | % | N | % | N | % | N | % |
| <NA> Participant left survey | 14 | 4 | 4 | 4 | 2 | 2 | 0 | 0 | 1 | 4 | 7 | 8 |
| -99 No response | 32 | 10 | 11 | 12 | 5 | 6 | 3 | 8 | 3 | 11 | 10 | 12 |
| 1 Never acceptable to edit the report | 93 | 29 | 32 | 36 | 19 | 24 | 6 | 15 | 6 | 21 | 30 | 36 |
| 2 Acceptable to edit the report without reviewer's permission | 109 | 34 | 14 | 16 | 37 | 46 | 27 | 68 | 11 | 39 | 20 | 24 |
| 3 Acceptable to edit the report, but only with reviewer's permission | 74 | 23 | 29 | 32 | 17 | 21 | 4 | 10 | 7 | 25 | 17 | 20 |
|  | 322 |  | 90 |  | 80 |  | 40 |  | 28 |  | 84 |  |

## Question A8_2 - When would an editor at your journal be permitted to edit a reviewer's report?

Situation 2: When the reviewer has used inappropriate or offensive language.

|  | All | | Ecology | | Economics | | Medicine | | Physics | | Psychology | |
| --- | --- | --- | --- | --- | --- | --- | --- | --- | --- | --- | --- | --- |
|  | N | % | N | % | N | % | N | % | N | % | N | % |
| <NA> Participant left survey | 14 | 4 | 4 | 4 | 2 | 2 | 0 | 0 | 1 | 4 | 7 | 8 |
| -99 No response | 17 | 5 | 3 | 3 | 6 | 8 | 2 | 5 | 0 | 0 | 6 | 7 |
| 1 Never acceptable to edit the report | 44 | 14 | 17 | 19 | 18 | 22 | 0 | 0 | 2 | 7 | 7 | 8 |
| 2 Acceptable to edit the report without reviewer's permission | 170 | 53 | 43 | 48 | 33 | 41 | 35 | 88 | 16 | 57 | 43 | 51 |
| 3 Acceptable to edit the report, but only with reviewer's permission | 77 | 24 | 23 | 26 | 21 | 26 | 3 | 8 | 9 | 32 | 21 | 25 |
|  | 322 |  | 90 |  | 80 |  | 40 |  | 28 |  | 84 |  |

## Question A8_3 - When would an editor at your journal be permitted to edit a reviewer's report?

Situation 3: When the reviewer has made an inappropriate reference to an author's gender, nationality, institution, age etc.

|  | All | | Ecology | | Economics | | Medicine | | Physics | | Psychology | |
| --- | --- | --- | --- | --- | --- | --- | --- | --- | --- | --- | --- | --- |
|  | N | % | N | % | N | % | N | % | N | % | N | % |
| <NA> Participant left survey | 14 | 4 | 4 | 4 | 2 | 2 | 0 | 0 | 1 | 4 | 7 | 8 |
| -99 No response | 18 | 6 | 4 | 4 | 5 | 6 | 2 | 5 | 0 | 0 | 7 | 8 |
| 1 Never acceptable to edit the report | 48 | 15 | 17 | 19 | 18 | 22 | 0 | 0 | 3 | 11 | 10 | 12 |
| 2 Acceptable to edit the report without reviewer's permission | 163 | 51 | 41 | 46 | 34 | 42 | 34 | 85 | 16 | 57 | 38 | 45 |
| 3 Acceptable to edit the report, but only with reviewer's permission | 79 | 25 | 24 | 27 | 21 | 26 | 4 | 10 | 8 | 29 | 22 | 26 |
|  | 322 |  | 90 |  | 80 |  | 40 |  | 28 |  | 84 |  |

## Question A8_4 - When would an editor at your journal be permitted to edit a reviewer's report?

Situation 4: When there are spelling and/or grammatical errors.

|  | All | | Ecology | | Economics | | Medicine | | Physics | | Psychology | |
| --- | --- | --- | --- | --- | --- | --- | --- | --- | --- | --- | --- | --- |
|  | N | % | N | % | N | % | N | % | N | % | N | % |
| <NA> Participant left survey | 14 | 4 | 4 | 4 | 2 | 2 | 0 | 0 | 1 | 4 | 7 | 8 |
| -99 No response | 14 | 4 | 4 | 4 | 4 | 5 | 1 | 2 | 0 | 0 | 5 | 6 |
| 1 Never acceptable to edit the report | 104 | 32 | 25 | 28 | 33 | 41 | 9 | 22 | 8 | 29 | 29 | 35 |
| 2 Acceptable to edit the report without reviewer's permission | 141 | 44 | 36 | 40 | 28 | 35 | 28 | 70 | 16 | 57 | 33 | 39 |
| 3 Acceptable to edit the report, but only with reviewer's permission | 49 | 15 | 21 | 23 | 13 | 16 | 2 | 5 | 3 | 11 | 10 | 12 |
|  | 322 |  | 90 |  | 80 |  | 40 |  | 28 |  | 84 |  |

## Question A8_5 - When would an editor at your journal be permitted to edit a reviewer's report?

Situation 5: When the review has English language problems.

|  | All | | Ecology | | Economics | | Medicine | | Physics | | Psychology | |
| --- | --- | --- | --- | --- | --- | --- | --- | --- | --- | --- | --- | --- |
|  | N | % | N | % | N | % | N | % | N | % | N | % |
| <NA> Participant left survey | 14 | 4 | 4 | 4 | 2 | 2 | 0 | 0 | 1 | 4 | 7 | 8 |
| -99 No response | 16 | 5 | 4 | 4 | 4 | 5 | 2 | 5 | 0 | 0 | 6 | 7 |
| 1 Never acceptable to edit the report | 95 | 30 | 22 | 24 | 30 | 38 | 8 | 20 | 8 | 29 | 27 | 32 |
| 2 Acceptable to edit the report without reviewer's permission | 124 | 39 | 35 | 39 | 24 | 30 | 26 | 65 | 14 | 50 | 25 | 30 |
| 3 Acceptable to edit the report, but only with reviewer's permission | 73 | 23 | 25 | 28 | 20 | 25 | 4 | 10 | 5 | 18 | 19 | 23 |
|  | 322 |  | 90 |  | 80 |  | 40 |  | 28 |  | 84 |  |

## Question A8_6 - When would an editor at your journal be permitted to edit a reviewer's report?

Situation 6: When the reviewer has left in their comments to the editor.

|  | All | | Ecology | | Economics | | Medicine | | Physics | | Psychology | |
| --- | --- | --- | --- | --- | --- | --- | --- | --- | --- | --- | --- | --- |
|  | N | % | N | % | N | % | N | % | N | % | N | % |
| <NA> Participant left survey | 14 | 4 | 4 | 4 | 2 | 2 | 0 | 0 | 1 | 4 | 7 | 8 |
| -99 No response | 18 | 6 | 7 | 8 | 5 | 6 | 1 | 2 | 1 | 4 | 4 | 5 |
| 1 Never acceptable to edit the report | 50 | 16 | 14 | 16 | 21 | 26 | 4 | 10 | 3 | 11 | 8 | 10 |
| 2 Acceptable to edit the report without reviewer's permission | 179 | 56 | 41 | 46 | 36 | 45 | 31 | 78 | 19 | 68 | 52 | 62 |
| 3 Acceptable to edit the report, but only with reviewer's permission | 61 | 19 | 24 | 27 | 16 | 20 | 4 | 10 | 4 | 14 | 13 | 15 |
|  | 322 |  | 90 |  | 80 |  | 40 |  | 28 |  | 84 |  |

## Question A8_7 - When would an editor at your journal be permitted to edit a reviewer's report?

Situation 7: When the editor disagrees with the reviewer's recommendation

|  | All | | Ecology | | Economics | | Medicine | | Physics | | Psychology | |
| --- | --- | --- | --- | --- | --- | --- | --- | --- | --- | --- | --- | --- |
|  | N | % | N | % | N | % | N | % | N | % | N | % |
| <NA> Participant left survey | 14 | 4 | 4 | 4 | 2 | 2 | 0 | 0 | 1 | 4 | 7 | 8 |
| -99 No response | 15 | 5 | 4 | 4 | 4 | 5 | 2 | 5 | 0 | 0 | 5 | 6 |
| 1 Never acceptable to edit the report | 238 | 74 | 67 | 74 | 64 | 80 | 25 | 62 | 17 | 61 | 65 | 77 |
| 2 Acceptable to edit the report without reviewer's permission | 22 | 7 | 5 | 6 | 1 | 1 | 8 | 20 | 5 | 18 | 3 | 4 |
| 3 Acceptable to edit the report, but only with reviewer's permission | 33 | 10 | 10 | 11 | 9 | 11 | 5 | 12 | 5 | 18 | 4 | 5 |
|  | 322 |  | 90 |  | 80 |  | 40 |  | 28 |  | 84 |  |

## Question A10 - Does your journal have an official policy on editing peer review reports?

|  | All | | Ecology | | Economics | | Medicine | | Physics | | Psychology | |
| --- | --- | --- | --- | --- | --- | --- | --- | --- | --- | --- | --- | --- |
|  | N | % | N | % | N | % | N | % | N | % | N | % |
| <NA> Participant left survey | 14 | 4 | 4 | 4 | 2 | 2 | 0 | 0 | 1 | 4 | 7 | 8 |
| -99 No response | 1 | 0 | 0 | 0 | 1 | 1 | 0 | 0 | 0 | 0 | 0 | 0 |
| 0 No | 258 | 80 | 71 | 79 | 67 | 84 | 34 | 85 | 21 | 75 | 65 | 77 |
| 1 Yes | 25 | 8 | 7 | 8 | 4 | 5 | 3 | 8 | 3 | 11 | 8 | 10 |
| 2 Unsure | 24 | 7 | 8 | 9 | 6 | 8 | 3 | 8 | 3 | 11 | 4 | 5 |
|  | 322 |  | 90 |  | 80 |  | 40 |  | 28 |  | 84 |  |

## Question A11 - Does your journal make any of the following documents available to the readership alongside the published article?

|  | All | | Ecology | | Economics | | Medicine | | Physics | | Psychology | |
| --- | --- | --- | --- | --- | --- | --- | --- | --- | --- | --- | --- | --- |
|  | N | % | N | % | N | % | N | % | N | % | N | % |
| <NA> Participant left survey | 18 | - | 5 | - | 5 | - | 0 | - | 1 | - | 7 | - |
| -99 No response | 6 | - | 2 | - | 2 | - | 1 | - | 0 | - | 1 | - |
| 0 None | 282 | 95 | 81 | 98 | 69 | 95 | 38 | 97 | 26 | 96 | 68 | 89 |
| 1 Unsigned peer reviewer reports | 3 | 1 | 1 | 1 | 0 | 0 | 0 | 0 | 1 | 4 | 1 | 1 |
| 2 Signed peer review reports | 2 | 1 | 1 | 1 | 0 | 0 | 1 | 3 | 0 | 0 | 0 | 0 |
| 3 Author responses | 4 | 1 | 1 | 1 | 1 | 1 | 1 | 3 | 1 | 4 | 0 | 0 |
| 4 Editorial decision letters | 5 | 2 | 1 | 1 | 1 | 1 | 1 | 3 | 1 | 4 | 1 | 1 |
| 5 I don't know | 3 | 1 | 1 | 1 | 1 | 1 | 0 | 0 | 0 | 0 | 1 | 1 |
| 6 Other | 8 | 3 | 0 | 0 | 2 | 3 | 0 | 0 | 0 | 0 | 6 | 8 |
| Total responses | 298 | - | 83 | - | 73 | - | 39 | - | 27 | - | 76 | - |

*Multiple answers possible - Percentages will not add up to 100%

## Question A12_R - Upon completion of peer review, are peer review reports provided to peer reviewers?*

|  | All | | Ecology | | Economics | | Medicine | | Physics | | Psychology | |
| --- | --- | --- | --- | --- | --- | --- | --- | --- | --- | --- | --- | --- |
|  | N | % | N | % | N | % | N | % | N | % | N | % |
| <NA> Participant left survey | 18 | 6 | 5 | 6 | 5 | 6 | 0 | 0 | 1 | 4 | 7 | 8 |
| -99 No response | 51 | 16 | 14 | 16 | 18 | 22 | 4 | 10 | 10 | 36 | 5 | 6 |
| 1 Shared | 199 | 62 | 53 | 59 | 37 | 46 | 32 | 80 | 8 | 29 | 69 | 82 |
| 2 Not shared | 39 | 12 | 12 | 13 | 14 | 18 | 3 | 8 | 8 | 29 | 2 | 2 |
| 3 I don’t know | 5 | 2 | 3 | 3 | 1 | 1 | 1 | 2 | 0 | 0 | 0 | 0 |
| 4 Other | 10 | 3 | 3 | 3 | 5 | 6 | 0 | 0 | 1 | 4 | 1 | 1 |
|  | 322 |  | 90 |  | 80 |  | 40 |  | 28 |  | 84 |  |

*Refer to “PRS_Results_Code.R” script for details on how this information was generated

## Question A12_E - Upon completion of peer review, are editorial decision letters provided to peer reviewers?*

|  | All | | Ecology | | Economics | | Medicine | | Physics | | Psychology | |
| --- | --- | --- | --- | --- | --- | --- | --- | --- | --- | --- | --- | --- |
|  | N | % | N | % | N | % | N | % | N | % | N | % |
| <NA> Participant left survey | 18 | 6 | 5 | 6 | 5 | 6 | 0 | 0 | 1 | 4 | 7 | 8 |
| -99 No response | 21 | 7 | 4 | 4 | 8 | 10 | 4 | 10 | 1 | 4 | 4 | 5 |
| 1 Shared | 233 | 72 | 63 | 70 | 47 | 59 | 34 | 85 | 21 | 75 | 68 | 81 |
| 2 Not shared | 35 | 11 | 11 | 12 | 15 | 19 | 2 | 5 | 3 | 11 | 4 | 5 |
| 3 I don’t know | 4 | 1 | 3 | 3 | 1 | 1 | 0 | 0 | 0 | 0 | 0 | 0 |
| 4 Other | 11 | 3 | 4 | 4 | 4 | 5 | 0 | 0 | 2 | 7 | 1 | 1 |
|  | 322 |  | 90 |  | 80 |  | 40 |  | 28 |  | 84 |  |

*Refer to “PRS_Results_Code.R” script for details on how this information was generated

## Question A13_D - What is the journal’s current policy on the availability of research data following publication?*

|  | All | | Ecology | | Economics | | Medicine | | Physics | | Psychology | |
| --- | --- | --- | --- | --- | --- | --- | --- | --- | --- | --- | --- | --- |
|  | N | % | N | % | N | % | N | % | N | % | N | % |
| <NA> Participant left survey | 18 | - | 5 | - | 5 | - | 0 | - | 1 | - | 7 | - |
| -99 No response | 10 | - | 1 | - | 3 | - | 1 | - | 2 | - | 3 | - |
| 0 Encourages authors to share | 168 | 57 | 41 | 49 | 37 | 51 | 18 | 46 | 17 | 68 | 55 | 74 |
| 1 Must make available post-publication if requested | 41 | 14 | 12 | 14 | 19 | 26 | 6 | 15 | 1 | 4 | 3 | 4 |
| 2 Requires an in-text statement | 52 | 18 | 20 | 24 | 8 | 11 | 11 | 28 | 2 | 8 | 11 | 15 |
| 3 Requires posting to a trusted repository | 34 | 12 | 17 | 20 | 10 | 14 | 6 | 15 | 0 | 0 | 1 | 1 |
| 4 No policy | 65 | 22 | 20 | 24 | 16 | 22 | 10 | 26 | 5 | 20 | 14 | 19 |
| 5 Not applicable | 10 | 3 | 2 | 2 | 0 | 0 | 5 | 13 | 2 | 8 | 1 | 1 |
| 6 I don't know | 4 | 1 | 1 | 1 | 0 | 0 | 1 | 3 | 1 | 4 | 1 | 1 |
| 7 Other | 8 | 3 | 1 | 1 | 2 | 3 | 1 | 3 | 1 | 4 | 3 | 4 |
| Total responses | 294 | - | 84 | - | 72 | - | 39 | - | 25 | - | 74 | - |

^Multiple answers possible - Percentages will not add up to 100%

*Refer to “PRS_Results_Code.R” script for details on how this information was generated

## Question A13_M - What is the journal’s current policy on the availability of research materials following publication?*

|  | All | | Ecology | | Economics | | Medicine | | Physics | | Psychology | |
| --- | --- | --- | --- | --- | --- | --- | --- | --- | --- | --- | --- | --- |
|  | N | % | N | % | N | % | N | % | N | % | N | % |
| <NA> Participant left survey | 18 | - | 5 | - | 5 | - | 0 | - | 1 | - | 7 | - |
| -99 No response | 40 | - | 9 | - | 15 | - | 4 | - | 5 | - | 7 | - |
| 0 Encourages authors to share | 143 | 54 | 37 | 49 | 28 | 47 | 15 | 42 | 13 | 59 | 50 | 71 |
| 1 Must make available post-publication if requested | 29 | 11 | 9 | 12 | 13 | 22 | 6 | 17 | 0 | 0 | 1 | 1 |
| 2 Requires an in-text statement | 34 | 13 | 16 | 21 | 4 | 7 | 6 | 17 | 0 | 0 | 8 | 11 |
| 3 Requires posting to a trusted repository | 16 | 6 | 6 | 8 | 6 | 10 | 3 | 8 | 0 | 0 | 1 | 1 |
| 4 No policy | 69 | 26 | 23 | 30 | 17 | 28 | 8 | 22 | 6 | 27 | 15 | 21 |
| 5 Not applicable | 13 | 5 | 3 | 4 | 1 | 2 | 6 | 17 | 2 | 9 | 1 | 1 |
| 6 I don't know | 4 | 2 | 1 | 1 | 0 | 0 | 1 | 3 | 1 | 5 | 1 | 1 |
| 7 Other | 7 | 3 | 2 | 3 | 2 | 3 | 0 | 0 | 1 | 5 | 2 | 3 |
| Total responses | 264 | - | 76 | - | 60 | - | 36 | - | 22 | - | 70 | - |

^Multiple answers possible - Percentages will not add up to 100%

*Refer to “PRS_Results_Code.R” script for details on how this information was generated

## Question A13_C - What is the journal’s current policy on the availability of research code following publication?*

|  | All | | Ecology | | Economics | | Medicine | | Physics | | Psychology | |
| --- | --- | --- | --- | --- | --- | --- | --- | --- | --- | --- | --- | --- |
|  | N | % | N | % | N | % | N | % | N | % | N | % |
| <NA> Participant left survey | 18 | - | 5 | - | 5 | - | 0 | - | 1 | - | 7 | - |
| -99 No response | 49 | - | 17 | - | 8 | - | 9 | - | 6 | - | 9 | - |
| 0 Encourages authors to share | 133 | 52 | 35 | 51 | 28 | 42 | 12 | 39 | 12 | 57 | 46 | 68 |
| 1 Must make available post-publication if requested | 32 | 13 | 9 | 13 | 16 | 24 | 4 | 13 | 1 | 5 | 2 | 3 |
| 2 Requires an in-text statement | 29 | 11 | 12 | 18 | 4 | 6 | 5 | 16 | 0 | 0 | 8 | 12 |
| 3 Requires posting to a trusted repository | 19 | 7 | 6 | 9 | 9 | 13 | 3 | 10 | 0 | 0 | 1 | 1 |
| 4 No policy | 65 | 25 | 17 | 25 | 19 | 28 | 8 | 26 | 6 | 29 | 15 | 22 |
| 5 Not applicable | 11 | 4 | 1 | 1 | 0 | 0 | 6 | 19 | 2 | 10 | 2 | 3 |
| 6 I don't know | 6 | 2 | 2 | 3 | 0 | 0 | 2 | 6 | 1 | 5 | 1 | 1 |
| 7 Other | 7 | 3 | 2 | 3 | 2 | 3 | 0 | 0 | 1 | 5 | 2 | 3 |
| Total responses | 255 | - | 68 | - | 67 | - | 31 | - | 21 | - | 68 | - |

^Multiple answers possible - Percentages will not add up to 100%

*Refer to “PRS_Results_Code.R” script for details on how this information was generated
